# Chemical Transformation of Humic Acid Molecules under the Influence of Mineral, Fungal and Bacterial Fertilization in the Context of the Agricultural Use of Degraded Soils

**DOI:** 10.3390/molecules26164921

**Published:** 2021-08-13

**Authors:** Patrycja Boguta, Kamil Skic, Zofia Sokołowska, Magdalena Frąc, Lidia Sas-Paszt

**Affiliations:** 1 Institute of Agrophysics, Polish Academy of Sciences, Doświadczalna 4, 20-290 Lublin, Poland; k.skic@ipan.lublin.pl (K.S.); sokolows@ipan.lublin.pl (Z.S.); m.frac@ipan.lublin.pl (M.F.); 2 Research Institute of Horticulture, Pomologiczna 18, 96-100 Skierniewice, Poland; lidia.sas@inhort.pl

**Keywords:** structure of humic acids, humification mechanism, fungal effect on humification, bacterial effect on humification

## Abstract

The main goal of this work was to study the structural transformation of humic acids (HAs) under the influence of selected strains of fungi (*Aspergillus niger* and *Paecilomyces lilacinus*) and bacteria (*Bacillus* sp., *Paenibacillus polymyxa* and *Bacillus amyloliquefaciens*) with/without the presence of NPK fertilizers. Two-year experiments were conducted on two different soils and HAs isolated from these soils were examined for structure, humification degree, and quantity using fluorescence and UV-Vis spectroscopy, elemental analysis, and extraction methods. Results showed that the applied additives contributed to the beneficial transformation of HAs, but effects differed for various soils. HAs from silty soil with higher organic carbon content showed simplification of their structure, and decreases in humification, molecular weight, and aromaticity under the influence of fungi and bacteria without NPK, and with NPK alone. With both fungi and NPK, increases in O/H and O/C atomic ratios indicated an increase in the number of O-containing functional groups. HAs from sandy soil did not show as many significant changes as did those from silty soil. Sandy soil exhibited a strong decline in HA content in the second year that was reduced/neutralized by the presence of fungi, bacteria, and NPK. Periodically observed fluorescence at ~300 nm/450 nm reflected formation of low-molecular HAs originating from the activity of microorganisms.

## 1. Introduction

Humic acids (HAs) belong to heterogeneous mixtures of polydispersed organic substances formed during the complex, biochemical processes of decomposition and transformation of organic matter [1]. These acids are valuable components of soil organic matter (SOM) known as long-lived sequesters of carbon [2,3], which show a great ability to improve cation exchange and buffer capacity [4], viscosity, compaction and air–water relations of soil [5]. HAs take part in the regulation of oxidation–reduction processes [6] and in the control of bioavailability, mobility, and chemical speciation of micronutrients [7,8]. This group of compounds is also a key source of energy for the activity of microflora and fauna in soils [9].

As HAs comprise a fraction of humic substances intermediate between fully soluble fulvic acids and non-soluble humins [10], they are extremely interesting from a chemical point of view. They undergo constant biochemical changes, the mechanisms and dynamics of which remain poorly understood. These complex transformations, known as “humification”, are manifested in an undefined structure with high elemental variability of carbon (C; 52–62%), hydrogen (H; 3–5.5%, nitrogen (N; 3.5–5%), and oxygen (O; 30–33%) [11], and in molecular weights ranging from 2000 to 50,000 Da [12]. Further difficulties in explaining the mechanism of HA transformation result from the fact that the direction of these changes may be influenced by numerous factors, including pH, ionic strength, presence of other chemical compounds [13], temperature, moisture, agronomic practices [14,15], and by the various contributions of the competitive mineralization processes.

The activity of microorganisms appears to be one of the most important factors inducing reorganization of HA structure. There is, however, little comprehensive information concerning the changes in the structure and humification process of HAs, as affected by fungal and bacterial strains with the simultaneous presence of mineral fertilization. Some studies have shown that selected fungal species can decolorize HAs [16], change their fluorophores [17], decompose HAs to low-molecular compounds of higher heterogeneity [18,19], or produce dark-brown polymers [20] with a proportion of elements such as H or N and functional groups like phenolic, carboxylic, or alcoholic different than the HAs of the soil. [21]. Considering bacteria, a few studies have indicated that depending on the strain, HAs may be quite resistant to degradation, but a slight modification of carbohydrate and carboxyl content can be observed [22]. The structure of HAs has also been reported to be broken down into smaller moieties in the presence of this kind of microorganisms [23]. In turn, another experiment confirmed the processes of formation of bacterial HAs with a lower degree of aromaticity, lower O content, and enrichment of N-functional groups [24]. Some reports have also indicated that the influence of microorganisms is governed by the interaction of HAs with minerals [14]. HAs located closely to mineral surfaces are protected from the microbial community, whereas external HAs are exposed to microbial attack [25]. Slower degradation of HAs may be linked with a higher hydrophobicity, as well as a higher number of long aliphatic chains and aromatic units [26,27]. In the context of mineral fertilization, some reports have shown that such supplementation may promote an increase in aliphatic groups in respect to aromatic moieties [28], an increase in N content [29], and a less complex structure for HAs [30].

Taking into account the above reports, it should be emphasized that the issue of structural changes in HAs under the influence of microorganisms and mineral fertilizers remains still very important. Complicated processes determining the mechanisms, dynamics, and direction of HA transformations suggest the need for new research concerning this scientific gap, especially in the context of the progressive loss of organic carbon in agricultural soils [31]. A lack of understanding regarding the effect of mineral additives and microorganism inoculation into the soil could also be the main limitation to the preparation of modern biofertilizers for agronomic use. Indeed, the concept of implementing biofertilizers is promising, based on several reports on the quality and quantity of crops [32], as well as quality of soils [33,34].

In view of the above problems, the main objective of this work was to identify possible changes in HAs obtained from two degraded arable soils under different treatments of selected bacterial and fungal strains, and mineral fertilization. For this purpose, the complementary spectroscopic methods UV-Vis and fluorescence spectroscopy, as well as elemental analysis, were used to determine and discuss the effect of the above-mentioned additives on the humification, structure, elemental composition, and content of HAs. Our results provide valuable information concerning the formation of new resources of humus substances. We believe that understanding the mechanisms governing the processes of HA formation and transformation can help to optimize the unfavorable phenomena of organic matter loss in the soil.

## 2. Results and Discussion

### 2.1. Structural Properties of HAs before the Addition of Fertilizers and Microorganisms

The structural parameters of HAs collected from Abruptic Luvisol (AL) and Brunic Arenosol (BA) soils at the beginning of the experiments (before any fertilization) are presented in Table 1.

Results showed that the HA content in both soils was approximately 0.3% d.m., which indicates a low content of these components in the studied soils [35]. The values of the E4/E6 parameter were similar and relatively high (6.12 and 6.43 for HAs in AL and BA soils, respectively), indicating the dominance of HAs with low molecular weight and relatively young age [36]. The Kumada parameter of humification, ΔlgK, based on the activity of chromophores, suggested that the studied HAs were B-type (range from Kumada’s classification: 0.6–0.8), and, thus, were moderately humified. The HIX parameter, which described the degree of humification on the basis of fluorescent moieties [37], was slightly lower for AL soil, indicating a prevalence of low molecular weight compounds with lower aromaticity, as compared to HAs in BA soil. The differences in values of HIX suggested the advisability and potential of applying fluorescence spectroscopy to analyze structural changes under the influence of fertilizers and microorganisms. The E2/E6 parameter expressed the ratio of initially humified structures to strongly humified ones. Higher E2/E6 values for HAs derived from AL soil suggested a higher number of structures of low transformation, typical for lignins [38].

The content of particular elements was typical of so-called “younger humic acids”, which was expressed as relatively low C content (50.7 and 51.8%), as well as high H (5.45 and 5.44%) and O (37.5 and 37.1%) content in HAs from AL and BA soils, respectively. Similar values of O and H, as well as O/H and O/C atomic ratios, for HAs from both soils indicated a comparable, high content of carbohydrates and oxygen functional groups, including COOH and OH [38,39]. The O-containing structures are of particular importance, as they are responsible for the sorption of micro- and macronutrients essential for plants [40]. Comparable O/C values also suggested a similar degree of humification and aromatic condensation in the HA structure [41]. In turn, the N content was significantly higher for HAs originating from AL soil, showing that the loss of amino acid N in HAs from BA soil was higher [42].

The H/C value of ~1.26–1.29 indicated that HAs had a structure intermediate between highly condensed aromatic rings and paraffin. According to Van Krevelen, values of H/C between 0.7 and 1.5 reflect aromatic rings conjugated with aliphatic chains (up to 10 C atoms [43,44]). Significant differences were found in C/N atomic ratios, which were higher for HAs from BA soil. Lower values of this parameter indicate a greater degree of stabilization and microbial decomposition of HAs in soil [45]. According to Tan [46], C/N ratios between 10 and ~18 can be attributed to well-developed HAs. A higher C/N can also result from lower N content caused by geological processes.

### 2.2. Changes in Elemental Composition and Atomic Ratios of HAs from Soils Treated with Mineral and Microbiological Additives

**Figure 1:**
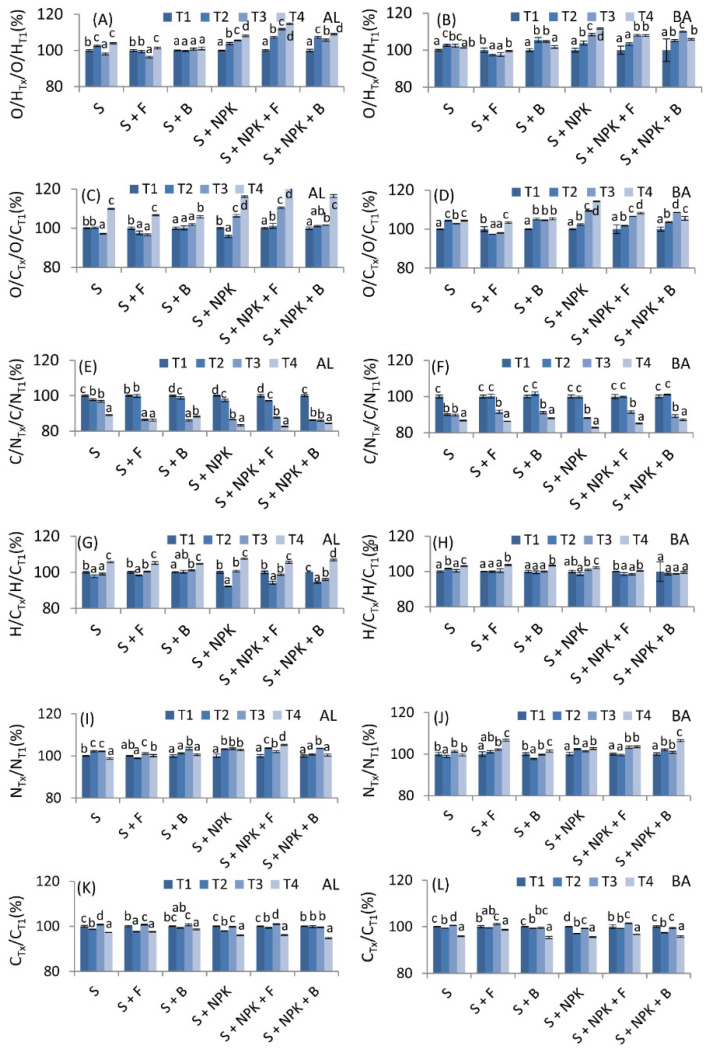
Changes in O/H (**A**,**B**), O/C (**C**,**D**), C/N (**E**,**F**), and H/C (**G**,**H**) atomic ratios, as well as the C (**I**,**J**) and N (**K**,**L**) content of HAs from Abruptic Luvisol (AL) and Brunic Arenosol (BA) soils, in the form of relative values with ±5% thresholds, marked in gray lines. The values measured at T2, T3, and T4 were compared to the T1 (Control, 100%) values of the plot. S—soil without additives, S + F—soil with fungal strains, S + B—soil with bacterial strains, S + NPK—soil with NPK, S + NPK + F—soil with fungal strains and NPK, S + NPK + B—soil with bacterial strains and NPK. The figure shows mean values ± standard deviation. The same small letters mean no significant differences between the values at the level of significance α = 0.05 (one-way ANOVA variance analysis, Tukey’s HSD test).

Elemental analysis allowed us to determine the atomic ratios that characterize the chemical structure of HAs. Changes in the O/H, O/C, C/N, and H/C ratios, as well as in the C and N content after addition of NPK or/and fungal (mix of *Aspergillus niger* and *Paecilomyces lilacinus)* or/and bacterial strains (mix of *Bacillus* sp., *Paenibacillus polymyxa* and *Bacillus amyloliquefaciens*), are shown in Figure 1 in the form of relative values with ±5% thresholds. The values measured at T2 (October, 1st year), T3 (April, 2nd year), and T4 (October, 2nd year) sampling dates were compared to the T1 (April, 1st year) values of the particular plot, to enable comparison among different variants and soils. Variants were marked for each soil as: S—soil without additives, S + F—soil with fungal strains, S + B—soil with bacterial strains, S + NPK—soil with NPK, S + NPK + F—soil with fungal strains and NPK, S + NPK + B—soil with bacterial strains and NPK. Additionally, graphical indicators of the significant increases/decreases in parameters in relation to the control variants, S (soil without additives) or S + NPK (soil with NPK), based on the calculations of percentage changes, are presented in Table 2.

Results indicated that the addition of bacterial strains without NPK (variants S + F, S + B) did not significantly affect the O/H ratio or did not provide a uniform trend for HAs derived from either of the studied soils during the two years of the experiment (Figure 1A,B, Table 2). In turn, fungal strains without NPK (S + F) caused decrease in O/H as compared to variant S of both soils. The addition of NPK alone increased the O/H ratio for HAs from both soils (stronger for BA soil), indicating that the formation of O-containing functional groups can be promoted in the presence of NPK. In the case of the variants with NPK, a positive effect of microorganisms on the O/H ratio in relation to the control variant (S + NPK) was observed with the addition of fungal strains to the AL soil (variant S + NPK + F; Figure 1A, Table 2). Particularly interesting and seemingly an adequate explanation of this phenomenon is a study by Ghosh et al. [13], which indicated that unhydrolyzed fungal HAs produced by *A. niger* could be characterized by more alcoholic OH groups than are found in unhydrolyzed soil HAs. Concerning our results, it can be concluded that the increase of O/H in the S + NPK + F variant could have resulted from the presence of a new pool of alcoholic OH groups. For HAs from BA soil, the increased O/H in the S + NPK + B and S + NPK + F treatments did not differ significantly in most sampling dates from that in soil without bacterial and fungal strains (S + NPK; Figure 1B).

Changes in O/C ratios were very similar to those of O/H ratios (Figure 1C,D). Although this ratio increased over the two years for most variants, the addition of fungal or bacterial strains to soils without NPK (S + F, S + B) did not cause significant changes in all sampling dates when compared to the variant without microorganisms (S). This suggests slight changes in the oxidation of the structure of HAs. The addition of NPK also caused an increase in the O/C ratio of HAs in the S + NPK, S + NPK + F and S + NPK + B variants. Similarly, as in the case of the O/H ratio, the addition of fungal strains to the AL soil with NPK significantly increased the O/C ratio in relation to the variant without fungi. In the case of the BA soil with the addition of fungi or bacteria (S + NPK + F, S + NPK + B), the increases in O/C were in general smaller as compared to the variant without microorganisms. Considering the relationship between O and C, it can be concluded that the increases in the O/C ratio in the abovementioned variants indicated an increase of carbohydrate and carboxylic contents, as well as a decrease in the degree of humification [41]. Such a trend was also connected with an increased number of OH phenolic groups, which could occur as a result of the transformation of organic substances into the “young HAs”. Humification occurring during the condensation of phenolic units is one of the most acceptable theories at present [21]. Additionally, according to See and Bronk [29], in the first stage of formation, humic substances could be more similar to fulvic acids, which have a structure characterized by relatively high O/C ratios. More aliphatic, simplistic, “younger” HAs were also found previously during NPK treatments when compared to unfertilized black soils [30].

Changes in C/N ratios (Figure 1E,F) were of great interest, dropping significantly and successively over the two years for HAs from both soils and in all fertilization variants, as well as in control soils. In the case of HAs from AL soil, the addition of NPK alone resulted in a faster and greater reduction of the C/N ratio in relation to HAs in soil without NPK. This was a rather surprising result, presumably due to the cumulative effect of a slight increase in N (Figure 1I,J) and a slight decrease in the C (Figure 1K,L) content of the HA structure. These two processes could indicate the first stage of humification, corresponding to the formation of lignin–nitrogen compounds [47]. A study by See and Bronk [29] that analyzed the humification process of *Spartina alterniflora* plants under laboratory conditions showed that a greater amount of N in humic substances is possible during humification when there is an abundance of NH_4_^+^ ions. The authors also reported that the C/N ratio of humics with low molecular weight decreased by more than half over the year of the experiment. We confirmed this trend in our studies over an even longer period of two years. Baddi et al. [48], when studying composting, reported that a decrease in C/N, as well as an increase in O/C, can also result from decreasing levels of C and increasing levels of O. In the case of composting, such changes can occur in as little as a few months, but in field experiments, more time may be needed. Indeed, our results showed clear structural changes in HAs during the humification process, as well as indicating the benefit of using the C/N ratio as a sensitive indicator to observe the above transformations. In most of analyzed cases, the addition of microorganisms did not significantly affect the C/N trend at each of its time points as compared to the variants without microorganisms but, interestingly, HAs from variants with fungal strains (both with and without NPK) were characterized by an increase in the amount of N. As shown in the literature, some fungal strains, including *A. niger*, can facilitate the transfer of inorganic N into organic N components of humic-like acid [49].

The H/C ratio significantly increased in the last year for HAs from AL soil (Figure 1G). This increase was mainly due to transformation over time, however, rather than the influence of NPK, or bacterial or fungal strains. The increasing trend of H/C reflected a gradual decrease in participation of condensed aromatic rings or substituted ring structures [41], meaning that structure tended to be simpler and aliphatic structures could have comprised a higher portion. The proportions of particular structures could change in the above way when the “young” HAs appeared in the HA pool. For BA soil, the dynamic of changes in this index were mostly not significant or showed no uniform trend (Figure 1H).

**Figure 2:**
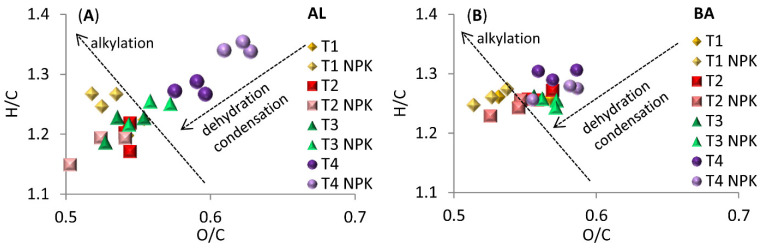
Van Krevelen diagrams for humic acids (HAs) from (**A**) Abruptic Luvisol (AL) and (**B**) Brunic Arenosol (BA) soils. Plots are designed to reveal changes over time of sampling (T1–T4) and differences among variants with or without NPK fertilization.

Interesting dependencies were revealed by Van Krevelen diagrams (Figure 2). According to Baranciková et al. [50], these plots can be considered a graphical-statistical representation of oxidation, dehydrogenation, dehydration, demethanation, and decarboxylation. Based on the characteristics of our samples, HAs collected at T4 from both soils exhibited a lower degree of condensation and dehydrogenation. In addition, for all NPK variants of AL soils, HA samples had a less condensed structure and a higher degree of hydrogenation than did HAs from soils without NPK. The results for HAs from various dates, as well as for HAs from soils without and with NPK, visibly grouped along a straight line, indicating pronounced changes in dehydration/hydration and condensation. Overall, it appeared that such structural changes were dominant in the HA structures.

### 2.3. Changes in Fluorescence, and Structural and Humification Parameters of HAs from Soils Treated with Mineral Fertilizers, and Fungal and Bacterial Strains

**Figure 3:**
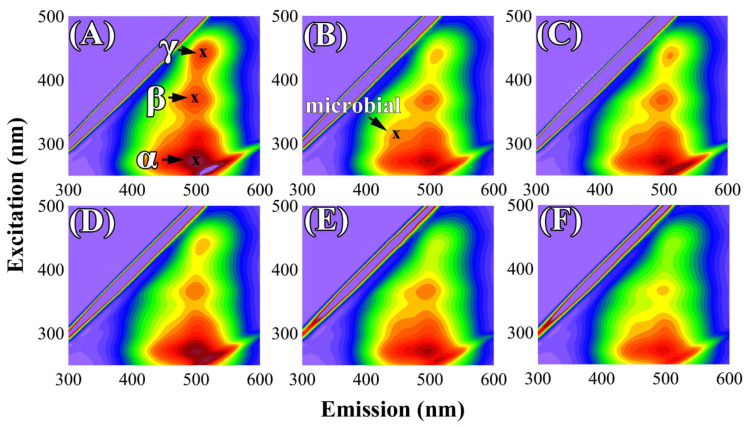
Exemplary EEM landscapes of humic acids (HAs) from different variants of soil samples collected in early April of the second year of experiments (T3): (**A**)—S + NPK from Brunic Arenosol (BA) soil; (**B**)—S + NPK + F from BA soil; (**C**)—S + NPK + B from BA soil; (**D**)—S + NPK from Abruptic Luvisol (AL) soil; (**E**)—S + NPK + F from AL soil; (**F**)—S + NPK + B from AL soil. S + NPK—soil with NPK, S + NPK + F—soil with fungal strains and NPK, S + NPK + B—soil with bacterial strains and NPK.

Fluorescence emission-excitation matrices (EEMs) were described by the coordinates and fluorescence intensities (FIs) of maxima that are commonly present in the EEM “landscapes” of humic substances [51]. Recorded spectra revealed three main groups of fluorophores in the HA samples, which were marked as α, β, and γ (Figure 3). The α maximum was located at ~270 nm/495 nm (ex/em). These fluorophores could be attributed to UV humic-like or fulvic-like systems [52,53]. The shift of the α maximum to a lower emission wavelength as compared to peaks β and γ could indicate a decrease in the number of aromatic rings condensed in a straight chain [54]. Furthermore, the shift of maximum to a shorter excitation wavelength could also be a valuable indicator of decreasing molecular weight, accompanied by an apparent increase in fluorescence efficiency [54]. The second group of fluorophores, marked as β, was placed in the region of ~370 nm/500 nm (ex/em) and was associated with visible humic-like compounds—groups of complex, poorly known fluorophores [52,55]. The γ fluorophores were located at ~435 nm/510 nm (ex/em). This signal can be assigned to terrestrial HAs derived from lignin with extended, highly polymerized, linear aromatic ring structures, in addition to other highly humidified macromolecules [56,57]. This result was in line with other studies showing that a maximum at higher ex/em wavelengths as compared to other peaks indicates more aromatic and humified macromolecules [58,59]. Exemplary EEM spectra are presented in Figure 3.

**Figure 4:**
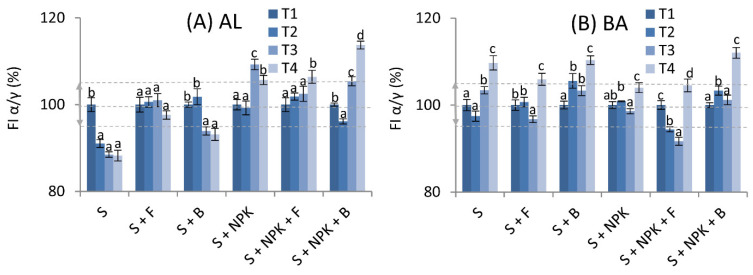
Changes in fluorescence intensity (FI) α/γ ratios in the form of relative values with ±5% thresholds marked in gray lines; (**A**)—humic acids (HAs) from Abruptic Luvisol (AL) soil, (**B**)—humic acids (HAs) from Brunic Arenosol (BA) soil. The values measured at T2, T3, and T4 were compared to the control (100%), marked as T1. S—soil without additives, S + F—soil with fungal strains, S + B—soil with bacterial strains, S + NPK—soil with NPK, S + NPK + F—soil with fungal strains and NPK, S + NPK + B—soil with bacterial strains and NPK. The figure shows mean values ± standard deviation. The same small letters mean no significant differences between the values at the level of significance α = 0.05 (one-way ANOVA variance analysis, Tukey’s HSD test).

The intensity of the maxima changed during the two years of the experiment. Understanding the reasons for these changes on the basis of one maximum, however, makes the correct interpretation difficult. For this reason, we took into account changes in the ratio of the fluorescence intensities of the α and γ maxima, which differed the most in terms of chemical structures. The changes in the FI α/γ ratio for the various variants during the two years of the experiment are shown in Figure 4 as relative values. The values at T2, T3, and T4 were compared to the T1 value (Control, 100%) of the particular plot. Additionally, graphical indicators of the increases/decreases of the FI α/γ ratio in relation to the control variants, S or S + NPK, based on calculations of percentage changes are presented in Table 2.

Changes in FI α/γ differed for the two tested soils. Statistical analysis showed that the parameter decreased over time for HAs from AL soil without additives (variant S), but the value of this decrease was significantly reduced for variants with fungi or bacteria without NPK (variant S + F and S + B) (Figure 4A). Indeed, it appears that the presence of fungi or bacteria increased the FI α/γ in comparison to that of HAs from soil without additives. Such results suggest a decrease in the number of structures with high molecular weight and higher aromaticity (Table 2). A decrease in aromatic structures in HAs was reported previously in topsoil as compared to subsoil horizon [60], which may confirm the beneficial effect of microorganisms whose activity occurs at a higher rate in the upper parts of soil. A similar effect was found in studies involving the transformation of lignite-type low-rank coal to HAs in the presence of bacteria consortia [24]. The HAs obtained in this manner were characterized as compounds with a lower degree of aromaticity, but higher hydrophilicity, N-functional group content, and aliphatic polar chains, as compared with HAs derived via traditional NaOH extraction. Interestingly, HAs generated with the three bacterial strains in those experiments exhibited high structural similarity. Previous studies have also shown that fungal strains of *A. niger* could be responsible for rapid degradation of plant litter [61]. Likely pathways for the formation of humic substances in the presence of microorganisms may result from “heteropolycondensation” catalyzed by microbial exoenzymes among small molecules (e.g., amino acids, phenols, and carbohydrates) released during enzymatic breakdown of biomacromolecules [62]. Alternatively, Řezáčová and Gryndler [17] showed that the presence of labile organic molecules (e.g., glucose) could affect the presence of blue or red shift in the fluorescence signal, indicating humification progress or regress. This indicates that the direction of HA transformation caused by fungal strains may depend on the presence of other substances and environmental conditions that determine the activity of microorganisms.

The addition of NPK alone to AL soil (variant S + NPK) caused a significant increase in the FI α/γ ratio over time, as compared to the variant without NPK (variant S). This observation is consistent with the results obtained in a study by De Mastro et al. [14], who reported that inorganic fertilization induced the formation of simpler and less aromatic molecules due to accelerated decomposition of organic substances, rather than their stabilization. Similarly, Galantini and Rosell [28] reported that HAs derived from soils fertilized with inorganic N and P compounds contained more aliphatic and phenolic groups than HAs from unfertilized soil. The influence of fungi and bacteria together with NPK was not clearly visible (except for the last date-insignificant changes for S + NPK + F and lack of uniform trend for S + NPK + B).

Concerning HAs from BA soil without NPK and microorganisms, a significant increase in FI α/γ ratios was observed over time, but the effect of adding bacteria or fungi was not clear in the case of S + F variant (lack of uniform trend in time). Similar results were observed for variants with NPK together with microbiological fertilization. The differences in the dynamics of HA changes in the two studied soils could be due to the differing recalcitrance of organic matter to microorganisms, as well as the mineralogical composition of the soils, which determines the presence of mineral–organic bonds of different strengths [63]. HA molecules complexed with strong reactive sites on mineral surfaces could be more protected against the microbial community [14,25]. In contrast, the external organic layers promote colonization by microorganisms [64] and may cause partial degradation and mineralization of HA molecules.

**Figure 5:**
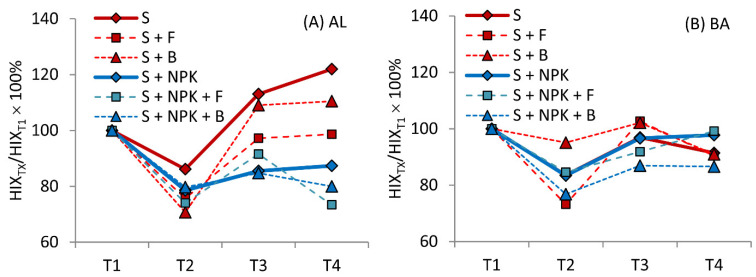
Changes in the humification index (HIX) for humic acids (HAs) from (**A**) Abruptic Luvisol (AL) and (**B**) Brunic Arenosol (BA) soils, expressed in the form of relative values. The values measured at T2, T3, and T4 were compared to the control values of the particular plot (100%) marked as T1 (Control, 100%). S—soil without additives, S + F—soil with fungal strains, S + B—soil with bacterial strains, S + NPK—soil with NPK, S + NPK + F—soil with fungal strains and NPK, S + NPK + B—soil with bacterial strains and NPK.

As part of a more detailed analysis, the fluorescent degree of humification, HIX, was determined. Results revealed an interesting phenomenon of seasonal changes in HIX, indicating a decrease (or stabilization) in its value in autumn periods (T2, T4) and an increase in spring periods (T1, T3; Figure 5). Decrease in HIX indicated the presence of higher number of low molecular compounds with lower aromaticity. Taking this into account, the decrease in HIX could be explained likely due to the increased amount of biomass or root exudates of maize in the soils. These substances could be transformed into fraction of “young HAs” decreasing HIX at T2 and T4.

The influence of microorganisms or NPK on HIX values in relation to the HIX of variants without microbial additives and without NPK is shown in Table 2. These results confirmed the conclusions based on the FI α/γ ratio: the addition of fungi or bacteria without NPK (S + F, S + B), as well as the addition of NPK only (S + NPK), significantly decreased the degree of humification of HAs from AL soil. Our findings are in line with previous reports that some fungal species are capable of decolorizing humic substances [16,65]. The significant and beneficial influence of *A. niger* in humification processes was also demonstrated in research concerning the formation of the humic bioactive compound “shilajit” [66]. Studies on composting have also shown that inoculation with *A. niger* reduces process time to 18 days, which was a more beneficial variant than other results [67]. Considering the results of our experiments, however, we believe that processes taking place in the soil do not have the same dynamics as compost processes. Instead, we observed an initial stage of HA formation, where low-molecular, poorly humified compounds were formed. Research by Wang et al. [49], conducted using cultures of cellulose and lignin with the addition of *A. niger*, showed that microbial residue from cellulose contained more aromatic compounds and fewer carbohydrates. In the case of lignin, more N-containing compounds, carbohydrates, and carboxylic acid derivatives, as well as less aromatic material, were found in the microbial residue. In those studies, the microbial residue from lignin tended to be simpler, with a lower degree of condensation and a higher degree of oxidation. It can, therefore, be assumed that the strains of fungi, under the conditions of our experiments, contributed predominantly to the transformation of lignin. Thus, our results indicate that fungal strains could have favorable properties for the decomposition and re-synthesis of organic matter. In the case of BA soil, only a significant decrease in the degree of humification was observed with the simultaneous addition of NPK and bacteria (variant S + NPK + B).

Another interesting phenomenon was a local (not occurring at all dates) increase in FI in the region of ~300 nm/450 nm (ex/em). These changes could reflect newly formed, low-molecular HAs, which may have resulted from the activity of microorganisms [68,69]. The spectral location of this maximum can also be attributed to molecules more similar to fulvic acids [70]. The FI disturbances were possibly due to the exudates of soil microorganisms [17]. Furthermore, microorganisms may utilize or modify humic substances, which may change the fluorescence parameters. The temporary nature of this signal, however, is difficult to explain.

Generally, results of fluorescence analysis suggest that changes of HAs under microbial influence may originate from different processes: direct production of HAs precursors by microorganisms or effect of microorganisms on transformation of native, soil HAs. We suppose that both processes are possible. This is indicated by the results of the fluorescence analysis. The addition of microorganisms caused gradual changes in the intensity of the native HAs fluorescence peaks (signals present in HAs of control soils), which suggests transformation of native HAs. On the other hand, completely new groups of fluorophores at ~300 nm/450 nm (ex/em) appearing for microbiological variants, suggest the appearance of compounds of another (microbial) etiology in the HAs pool. Our assumptions are supported by some other studies. Transformation of existed, native HAs under microorganisms’ influence can be deduced from different reports showing among others: brokening the structure of HAs into smaller moieties in the presence of bacteria or fungi [18,19,23], decolorization of native HAs [16] or acceleration of transformation of organic matter and progress of maturation of formed HAs in composting processes [67]. In turn, production of HAs by microbial strains were reported in the context of fungal exudates [17,21].

### 2.4. Changes in Degree of Humification of HAs Derived from Soils with Mineral Fertilization, and Fungal and Bacterial Strains Based on UV-Vis Spectroscopy

**Figure 6:**
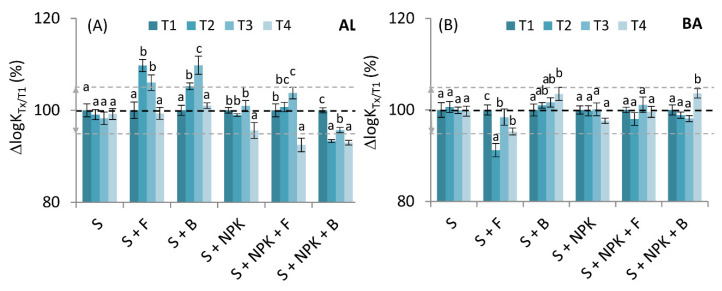
Changes in the Kumada degree of humification (ΔlgK) in the form of relative values with ±5% thresholds marked in gray lines; (**A**)—humic acids (HAs) from Abruptic Luvisol (AL) soil, (**B**)—humic acids (HAs) from Brunic Arenosol (BA) soil. The values measured at T2, T3, and T4 were compared to the control (100%) marked as T1. S—soil without additives, S + F—soil with fungal strains, S + B—soil with bacterial strains, S + NPK—soil with NPK, S + NPK + F—soil with fungal strains and NPK, S + NPK + B—soil with bacterial strains and NPK. The figure shows mean values ± standard deviation. The same, small letters mean no significant differences between the values at the level of significance α = 0.05 (one-way ANOVA variance analysis, Tukey’s HSD test).

Registration of the UV-Vis spectra for the examined HAs made it possible to determine the so-called Kumada degree of humification (ΔlgK) based on the relationships between strongly and poorly humified structures. The relative changes in this parameter are shown in Figure 6. The values at T2, T3, and T4 were compared to the value of T1 (control) of the particular plot. Additionally, graphical indicators of increases/decreases of a given parameter in relation to the control variants, S or S + NPK, are presented in Table 2.

Results for the AL soil showed that the value of this parameter increased significantly in the T2 and T3 due to the addition of fungal or bacterial strains without NPK, in comparison to HAs from soil without microorganisms. This direction of ΔlgK change indicated a decrease in the degree of humification [10]. A similar trend was also supported by our fluorescence studies. Statistically significant decreases in the values of ΔlgK for HAs from the S + NPK + B variant was quite interesting, as it was the only variant in which the addition of microorganisms caused an increase in the degree of humification in T2–T4 sampling times. The previously used instrumental methods did not show any clear direction of changes in the degree of humification for HAs from this variant, so these results require further, in-depth research.

The addition of microorganisms or NPK did not cause significant changes or a clear uniform trend during experiment period in HAs from BA soil, which likely resulted from the differences in the physicochemical properties of BA soil in relation to AL. This is in line with the conclusions of Mącik et al. [33] regarding the different effects of microbial communities on enzymatic activity, and the genetic and functional diversity of BA and AL soils.

### 2.5. Changes in the Content of HAs in AL and BA Soils

HA content in the soils under various fertilization treatments is presented in Table 3. The one-way ANOVA with the post-hoc tests showed that changes in this parameter over time were statistically significant at α = 0.05 but did not show a uniform trend. HA content appeared to decline in BA soil at T3 when compared to T1. This decline was less pronounced in the variants with fungi or bacteria, as well as with NPK alone, when compared to variants without microorganisms or NPK, respectively. In the case of AL soil, the amount of HAs decreased in T3 more in S soil than in S + NPK. The addition of fungi or bacteria in the variants with NPK also caused in T3 a statistically significant decrease in HAs content. This effect was stronger as compared to the S + NPK treatment. The HA levels of S + NPK + B treatments remained higher than in variants with bacteria without NPK.

The loss of HAs in all studied variants was associated with the differing dynamics of mineralization processes. Previous studies on various strains of bacteria have indicated that HAs may be degraded by some strains, thus, HA carbon can be mineralized by up to a few percent in four weeks [71]. The intensification of this process at T3 was likely due to the increased amount of biomass left in the field after the autumn harvest. The reduced loss of HA content in variants with NPK or with microorganisms observed at T3 (spring, second year of the experiment) in BA soil, in relation to control variants, could suggest a beneficial effect of fertilization on the transformation of corn straw remaining on topsoil after the corn harvest in the first year. Microorganisms supported the decomposition of organic matter to simpler compounds, obtaining energy and nutrients from the process [62], and making the resynthesis and formation of humic compounds possible. The periodic fluctuations observed in HA content suggest, however, the predominance of mineralization or humification at some periods.

### 2.6. Agronomic Implications and Further Research

Our results show that mineral and microbial additives may contribute to the beneficial transformation of HAs in the agricultural aspect. Effects of fertilization may differ, depending on soils’ physicochemical properties. In our research, such an effect was observed for AL soil, characterized by a high silt content (~60%) and a more than two times higher content of organic carbon than that in sandy BA soil. A decrease of HA humification in AL soil under the influence of NPK, fungi, or bacteria, may indicate the formation of new humic compounds from organic matter available in the soil. This was supported by the reduced loss of HA content in relation to the variant without NPK. The formation of a new pool of HAs under the influence of fungal or bacterial activity was not confirmed in our study by increasing amounts of HAs. In this case, the mineralization processes were likely intensified and could interfere with observation of the increasing HA content. The formation of humic compounds should, however, undoubtedly be considered a favorable process, preventing a drastic decrease in organic carbon in the soil. Humic compounds are more valuable than unprocessed organic matter in micronutrient management.

In addition, the increase in the O/C and O/H ratios in the S + NPK + F variant shows that the presence of fungi may contribute to increases in the amount of O-containing functional groups, which play an important role in the cation exchange and buffer capacity of soils, as well as the bioavailability of microelements. The addition of NPK also revealed a beneficial effect, inducing a decrease in the C/N ratio, which could be partly explained by the increase in N in the HA structure. In this case, despite its low abundance, N plays the role of a strong electron donor that forms strong bonds with microelements. Such complex compounds are likely very beneficial because they help to retain micro- and macro-elements in the soil, as well as prevent the elements from leaching into the deeper soil layers.

Interestingly, trends observed for HAs in AL soil were not always observed for HAs in BA soil. The BA soil, characterized by a high sand content (~87%; [72]), showed a strong decrease in HA content at T3 for every treatment. This decline was reduced/neutralized, however, in the variants with fungi (reduction of decline by 24% and 11% for S + F and S + NPK + F, respectively) and with bacteria (reduction of decline by 27% and 18% for S + B and S + NPK + B, respectively) in relation to the variants without microorganisms. Mineralization of HAs was also reduced by NPK (reduction of the decrease by 20%) when compared to the decrease in HA content in the soil without NPK. Contrary to HAs in AL soil, HAs in BA soil did not significantly change the structure or the degree of humification under the influence of microorganisms or mineral fertilizers. Our observations indicate the need for further research that considers a wider spectrum of soils. Formation and transformation of HAs may differ due to the different physicochemical properties of soil. Moreover, the large variability in the parameters describing humification could result from the method of soil tillage. Literature shows that no tillage induces the formation of more stable humic matter [14]. Thus, it could be concluded that conventional tillage (used in our experiment) could periodically disturb stability and balance. Destabilization of organic C in this case may be related to higher soil aeration, promoting the activity of microorganisms [14]. Moreover, transformation of organic matter in soil may depend on water potential, oxygen and nutrient supply, temperature, and pH because soil microorganisms’ survival and mobility are strongly dependent on these parameters [62]. Our research also indicates the need for long-term studies to evaluate the effect of microorganisms on the abundance of HAs. Large variations in this parameter show that various factors (e.g., seasonal changes) may distort the picture of this process and a short-term approach may not be sufficient to fully capture the changes.

It should be noted, however, that the strains of microorganisms proposed in our research show beneficial, biological effects in the soils tested, which was confirmed by our previous studies. Mącik et al. [33] observed the increase of amino acids metabolism in BA soil after addition of phosphorus fertilizers enriched with the same strains used in current study as compared to the control soil with mineral fertilization. The above authors showed also that mineral fertilization supplemented to the soil together with the bacterial strains provided positive effect on the microbiome changes and genetic biodiversity in soil. Walkiewicz et al. [72] studied the response of soil microbial biomass and activity to mineral fertilizers enriched with the same microorganisms as in our current study. Their results showed that applied biofertilizers increased the biological activity of soil, even in reduced doses of mineral additives. All above studies indicate indirectly on changes in the microbial community of studied BA and Al soils. It also suggested that applied microorganisms become an important part of the whole microbial community in the soils.

## 3. Materials and Methods

### 3.1. Location and Physicochemical Properties of Soils Used in Field Experiments

Field experiments were carried out in 2018 and 2019 on two mineral soils: Brunic Arenosol (BA), located in Biszcza (50°39′ N, 22°65′ E), and Abruptic Luvisol (AL), located in Basznia (50°15′ N, 23°27′ E). Some properties of the above soils have been presented previously by Pertile et al. [34] and Walkiewicz et al. [72]. Briefly, the BA sandy soil contained clay, silt, and sand at levels of 0.93%, 12.44%, and 86.63%, respectively, and was classified as soil degraded due to inappropriate cultivation and fertilization. The AL silty loam soil was characterized by 5.44%, 60.29%, and 34.37% content of clay, silt, and sand, respectively, and was chemically degraded due to sulfur extraction in the past [72]. The BA soil contained 1.39 mg kg^−1^ dry matter (d.m.) of N-NO_3_, 2.4 mg kg^−1^ d.m. of N-NH_4_, 2.9 mg 100 g^−1^ of K, and 17.4 mg 100 g^−1^ of P. In contrast, the AL soil contained 2.26 mg kg^−1^ d.m. of N-NO_3_ and 6.77 mg kg^−1^ d.m. of N-NH_4_, as well as 5.3 mg 100 g^−1^ of K and 4.8 mg 100 g^−1^ of P. The organic carbon content was higher in AL soil (3.76%) than in BA soil (1.54%), and the pH_KCl_ of BA soil was 4.8 whereas that of AL soil was 4.9 [33].

### 3.2. Selection of Mineral Fertilizers, and Fungal and Bacterial Strains

**Figure 7:**
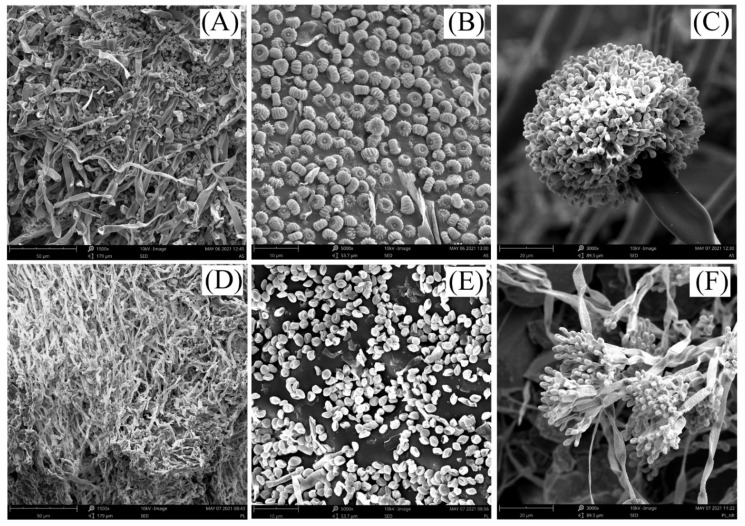
Scanning electron microscope (SEM) photographs showing structures of fungal strains growing on carriers: (**A**)—*Aspergillus niger* G119AA, mycelium with hyphae; (**B**)—*A. niger* G119AA, conidia; (**C**)—*A. niger* G119AA, conidiophore with conidial head; (**D**)—*Paecilomyces lilacinus* WT15A, mycelium with hyphae; (**E**)—*P. lilacinus* WT15A, conidia; (**F**)—*P. lilacinus* WT15A, conidiophores with phialides and conidia.

Fungal strains applied to soils included beneficial *Aspergillus niger* G119AA and *Paecilomyces lilacinus* WT15A. To obtain an equal concentration (fungal spore density) of both strains in the final biopreparation (1–3 × 10^7^ CFU per 1 g of biopreparation), two carriers—rice flour and corn flour—containing proliferated strains of *A. niger* G119AA and *P. lilacinus* WT15A were mixed before application at a ratio of 1:15, respectively [34]. The fungal strains used in the study showed no antagonism to each other (data not shown). Unpublished data of the Institute of Horticulture in Skierniewice indicated that joint application of the above strains shows biostimulatory and plant protective effects for horticultural plants. Moreover, Pertile et al. [34] indicated that the addition of the above-mentioned fungal strains mitigated the adverse effects of urea on the soil environment under maize cultivation. Bacterial strains included *Paenibacillus polymyxa* CHT114AB, *Bacillus amyloliquefaciens* AF75BB, and *Bacillus* sp. CZP4/4. All fungal and bacterial strains originated from the SYMBIOBANK collection of the Research Institute of Horticulture in Skierniewice, Poland. Mącik et al. [33] showed positive effect of the phosphorus mineral fertilizer enriched with the above-mentioned bacterial strains in maize crop on the enzymatic activity and microbial functional and genetic diversity of the soil. Exemplary microphotographs of the used fungal strains are shown in Figure 7. The images were captured using the scanning electron microscope Phenom ProX (Thermo Fisher Scientific INC., Waltham, MA, USA).

The NPK fertilizers used in the experiments were the following: Polifoska 6-NPK(S) 6-20-30-(7) (Group Azoty Company, Tarnów, Poland); Zaksan, which is granulated NH_4_NO_3_ with filler (calcium carbonate and magnesium carbonate; Group Azoty Company, Tarnów, Poland); and granulated potassium salt, 60% K_2_O (Bialchem, Białystok, Poland).

### 3.3. Experiment Design for Application of Mineral Fertilizers, Bacterial and Fungal Strains, and Soil Sampling

The AL and BA soils were used to perform two years of field experiments during maize cultivation (Hybrid P9241, Pioneer Hi-Bred Poland Sp. z o.o., Warsaw, Poland). The selected maize hybrid was characterized by a very high grain yield potential, drought tolerance, and stay-green properties, as well as high stalk and root strength, and grain dry-down properties.

Field experiments included six fertilization variants with three replicate plots (10 m × 15 m) for each treatment, for both AL and BA soils. Variant I (S) was left with no fertilization or microbial amendments. Variants II and III were soil without NPK but with fungal (S + F) or bacterial (S + B) strains, respectively. Variant IV received only NPK fertilization (S + NPK), and variants V and VI received NPK with the addition of fungal (S + NPK + F) or bacterial strains (S + NPK + B), respectively.

Doses of NPK fertilizers, as well as fungal and bacterial strains, were calculated for both soils by taking into account crop requirements and initial nutrient content, including N, P, and K. The detailed annual fertilization plan for both soils is presented in Table 4.

Briefly, variants of AL and BA soils with no NPK, or fungal or bacterial strains (S) were not treated with any amendments through the two years of the experiment. Variant II (S + F) was treated with fungal strains split into three doses per year: 17 + 7.5 + 6 kg per 1 ha for AL soil and 17 + 8 + 7 kg per 1 ha for BA soil. The first dose was related to pre-sowing (April), the second was applied during maize vegetation (May), and the third was applied in June. Variant III (S + B) for both soils included enrichment of soils with one dose of the bacterial strains (14 kg per ha) before sowing (April).

Variants IV, V, and VI (S + NPK, S + NPK + F, S + NPK + B) were supplemented with fungal or bacterial strains at the same time and in the same amounts as in the cases of variants without NPK. Different amounts of fungal strains and the same amounts of bacterial strains added to two studied soils resulted from the fact that the fungal and bacterial inoculum was intended to be used also in other experiments as enrichment of mineral fertilizers such as urea and phosphorus, respectively. Therefore, the amounts of fungal and bacterial addition were calculated based on the maize nutritional requirements and nutrients availability is both tested soils, taking into account production methods of biofertilizers described by Borowik et al. [73]. Mineral fertilizers containing NPK elements were applied in form of Polifoska 6, potassium salt, and ammonium nitrate (Table 4). Polifoska 6 was introduced in April, before sowing (250 or 300 kg per ha in BA or AL soils, respectively) and simultaneously with potassium salt (162 or 135 kg per ha in BA or AL soils, respectively). For the variants with microorganisms, this date coincided with the introduction of bacteria or first dose of fungi. Ammonium nitrate was split into two doses of 295 and 147 kg per ha (the same for both soils), applied as a top-dressing with a two-week interval. These dates corresponded to seven days before the second dose of fungi and seven days before third dose of fungi and were used in all NPK variants for comparison purposes. All fertilization plans were repeated in the second year on the same dates and at the same doses.

Soil samples were collected from a depth of 0–25 cm four times over the two years (two samplings each year). The first sampling (T1) was before the application of fertilizers, and fungal or bacterial strains (early April), and the second sampling (T2) was after the maize harvest but before plowing (October). Collection of samples T3 and T4 in the second year occurred on the same dates as in the first year. Each type of fertilization was arranged in three separate ecological replications with 10 m × 15 m plots for both soil types. Samples were taken from five randomly selected sites at each subplot, and thoroughly homogenized. After air drying, soils were sieved through a 2-mm mesh and stored in the dark.

### 3.4. Analysis of Structural Changes and Humification of Humic Acids during Treatment with Microorganisms and Fertilizers

Extraction of HAs was performed for all collected soil samples according to the procedure recommended by the International Humic Substances Society [74]. The effect of the addition of mineral fertilizers, and bacterial and fungal strains on structural changes and the degree of humification of HAs was analyzed with the use of several complementary methods, enabling the observation of changes at the molecular level.

The elemental analysis allowed the determination of changes in the composition of HAs, as well as determining the atomic relations characterizing the presence of specific chemical structures, relationships between them, and the degree of humification. All measurements were performed on freeze-dried HA samples using a CHNSO 2400 analyzer (Perkin Elmer, Waltham, MA, USA). The percentage content of C, N, H, O, and S was determined to allow the calculation of atomic ratios O/C, C/H, C/N, and O/H. Van Krevelen diagrams (H/C vs. O/C) were plotted to analyze structural changes in HAs under the influence of the studied fertilizers and microbiological amendments over the two years.

Fluorescence spectroscopy was used to observe structural changes at the molecular level. All measurements were performed using a Hitachi FL7000 apparatus (Hitachi, Tokio, Japan). Firstly, a 5-mg aliquot of each HA sample was dissolved in 50 mL of deionized water with the addition of a few drops of 1M NaOH. The solutions were then mixed using a magnetic stirrer in a N_2_ atmosphere until the HAs were completely dissolved. Next, prepared solutions were diluted twice with deionized water to obtain a final HA concentration of 50 mg dm^−3^, with 1M NaOH or 1M HCl used to adjust the final pH of the solutions to 7.0. Fluorescence measurements were performed in the 3D emission-excitation matrix (EEM) mode to fully visualize the changes taking place in the major groups of HA fluorophores. Wavelength emission was scanned from 300 to 600 nm while the excitation wavelength was increased sequentially by 5-nm steps from 250 to 500 nm. The EEM spectra were recorded with the scan speed fixed at 12,000 nm min^−1^, and emission and excitation slits set on 10 nm. EEM plots were generated as contour maps using the Surfer software (Golden Software Inc., Golden, CO). Fluorescence intensities (FI, arbitrary units) of three fluorescence peaks (designed as α, β and γ) were analyzed. From the obtained results, a humification index (HIX) was also calculated as the fluorescence intensity in the 300→345 nm region divided by the sum of the intensity in the 300→345 nm and 435→480 nm regions [37]:

HIX = FI(300–345)/[FI(300–345) + FI(435–480)](1)

UV-Vis spectroscopy was used to visualize molecular changes in the structures of chromophores. Samples of all HAs were prepared in the form of aqueous solutions, as described above. Measurements were performed using a Hitachi U-5100 spectrometer (Hitachi, Japan) in the range of 200–800 nm, with a scan speed of 400 nm min^−1^, a sampling interval of 2 nm and slit width fixed at 5 nm. From the obtained data, the degree of humification was calculated as the difference between the decimal logarithm of absorbance at 400 and 600 nm:

ΔlgK = lgA_400_ − lgA_600_ (2)

Additionally, for a more detailed characterization of the HAs at initial date of experiment, the parameters of E4/E6 (absorbance ratio at 465 nm and 665 nm) and E2/E6 (absorbance ratio at 280 nm and 665 nm) were determined from the UV-Vis data.

Three replicates were performed for each treatment and the results were averaged. The absolute values of all parameters for HAs from control soils (variant S at T1 date) are presented in Table 1 to show the initial properties of HAs. The changes in all the determined parameters of HAs during the two years of the experiment are expressed in the subsequent figures in relation to the value measured at T1, which, therefore, always had the value of 100%. The percentage expression of a given parameter made it possible to compare the dynamics of changes occurring between two different soils, as well as to avoid misleading conclusions resulting from spatial variability on the scale of the field.

The statistical analysis of the obtained data was performed using Statistica 13.1 (Dell Inc.). The one-way analysis of variance (ANOVA) with post-hoc analysis (HSD Tukey test) was performed to test the differences for measured parameters such as atomic ratios, fluorescence intensities UV-Vis results. The tests were carried out separately for each variant of fertilizer or sampling time. All measurements were in triplicate, and the significance level was estimated at α = 0.05. The presented results were provided with standard deviations. The assumption of normal distribution for all data used in statistical calculations was verified by the Shapiro–Wilk test.

## 4. Conclusions

The main goal of this study was to analyze the effects of mineral and microbiological fertilization on the content, structure, and humification of HAs from two soils with different physicochemical properties.

The results of our research showed that the structural parameters of HAs determined from the data of florescence spectrometry, UV-Vis spectrophotometry, and elemental analysis may change depending on the soil properties, type of additive and over time. A particularly pronounced effect of the addition of NPK and microorganisms was visible in the case of HAs of silty soil (~60% of silt, two times higher content of C_org._ than in sandy soil), for which humification parameters (FI_α/γ_, HIX, ΔlogK), and atomic, structural ratios (O/C, O/H) were strongly affected. For this soil, changes in HAs were mainly directed towards lowering degree of humification, amounts of aromatic structures, and molecular weight. What is more, results obtained in our study suggest that changes of HAs under microbial influence may originate from two processes: direct production of HAs precursors by microorganisms (fluorophores at ~300 nm/450 nm (ex/em)) and transformation of native, soil HAs (gradual changes in the intensity of fluorescence peaks originating from the native HAs).

The loss of HAs in all studied variants was associated with different dynamics of mineralization processes. The intensification or attenuation of this process at some experiment dates may be linked with fluctuating amounts of biomass. The reduced loss of HAs in variants with NPK or with microorganisms observed in the spring of the second year, in relation to control variants, could suggest a beneficial effect of fertilization on the transformation of corn straw.

It should be emphasized that, the observed differences for HAs of two different soils indicate the need for further research based on a greater spectrum of soils. As was shown, the formation and dynamics of HA transformation may vary due to differences in the physicochemical properties of soils, their mineralogical composition, and the recalcitrance of organic matter to microorganisms.

## Figures and Tables

**Table 1:** The content, structural, and chemical properties of humic acids (HAs) from Abruptic Luvisol (AL) and Brunic Arenosol (BA) untreated soils at the start of the field experiment. The table shows mean values ± standard deviation.

	HAs (%)	E4/E6	ΔlgK	E2/E6	HIX	C (%)	H (%)	N (%)	S (%)	O (%)	O/H	O/C	C/N	H/C
AL	0.30	6.12	0.74	38.2	21.7	50.7	5.45	5.02	1.40	37.5	0.43	0.55	11.8	1.29
	±0.02	±0.85	±0.03	±6.65	±1.13	±0.31	±0.07	±0.09	±0.03	±0.37	±0.01	±0.01	±0.20	±0.02
BA	0.29	6.43	0.72	36.7	23.3	51.8	5.44	4.54	1.12	37.1	0.43	0.54	13.3	1.26
	±0.02	±0.55	±0.02	±4.00	±2.12	±0.71	±0.08	±0.08	±0.04	±0.75	±0.01	±0.02	±0.30	±0.01

**Table 2:** Direction of changes in atomic ratios and parameters of humification processes in comparison to the control soil (S) or soil with only NPK fertilizer (S + NPK). Bold letters F, B, or NPK indicate an influencing factor. Analyses were performed in relation to the particular variants without influencing factors. For example, changes in AL variant S + NPK were compared with AL variant S.

Influencing Factor (Bold)	Compared to:	C/N	H/C	Humification as FI_α/γ_	Humification as HIX	Humification as ΔlogK	Humification as O/C	O/H
AL (S + **F**)	AL (S)	-	-	↓	↓	↓	-	↓
AL (S + **B**)	AL (S)	-	-	↓	↓	↓	-	-
AL (S + NPK **+ F**)	AL (S + NPK)	-	-	-	↓*	-	↓	↑
AL (S + NPK **+ B**)	AL (S + NPK)	-	-	-	-	↑	-	-
AL (S + **NPK**)	AL (S)	↓	-	↓	↓	-	↓*	↑*
BA (S + **F**)	BA (S)	-	-	-	-	↑*	↑*	↓
BA (S + **B**)	BA (S)	-	-	-	-	-	-	-
BA (S + NPK + **F**)	BA (S + NPK)	-	-	↑*	-	-	-	-
BA (S + NPK + **B**)	BA (S + NPK)	-	-	-	↓	-	-	-
BA (S + **NPK**)	BA (S)	-	-	↑*	-	-	↓*	↑

* Uncertain direction of changes, e.g., cases where a particular trend was disturbed on one of the dates. - No significant changes.

**Table 3:** Humic acid (HAs) content in Abruptic Luvisol (AL) and Brunic Arenosol (BA) soils at different sampling dates. The table shows mean values ± standard deviation. The same, small letters mean no significant differences between the values at the level of significance α = 0.05 (one-way ANOVA variance analysis, Tukey’s HSD test).

	% HAs in AL				% HAs in BA (%)			
	T1	T2	T3	T4	T1	T2	T3	T4
S	0.33 ^c^ ± 0.011	0.35 ^d^ ± 0.017	0.27 ^a^ ± 0.026	0.30 ^b^ ± 0.013	0.30 ^b^ ± 0.017	0.29 ^b^ ± 0.024	0.15 ^a^ ± 0.024	0.31 ^b^ ± 0.025
S + F	0.30 ^b^ ± 0.007	0.36 ^c^ ± 0.024	0.26 ^a^ ± 0.019	0.27 ^a^ ± 0.018	0.33 ^b^ ± 0.008	0.32 ^b^ ± 0.021	0.24 ^a^ ± 0.035	0.32 ^b^ ± 0.019
S + B	0.29 ^c^ ± 0.013	0.32 ^d^ ± 0.009	0.16 ^a^ ± 0.023	0.27 ^b^ ± 0.013	0.28 ^b^ ± 0.011	0.29 ^b^ ± 0.025	0.22 ^a^ ± 0.026	0.29 ^b^ ± 0.013
S + NPK	0.30 ^b^ ± 0.015	0.31 ^c^ ± 0.014	0.28 ^a^ ± 0.006	0.38 ^d^ ± 0.009	0.30 ^c^ ± 0.015	0.28 ^b^ ± 0.023	0.21 ^a^ ± 0.025	0.29 ^bc^ ± 0.019
S + NPK + F	0.27 ^b^ ± 0.012	0.29 ^c^ ± 0.013	0.23 ^a^ ± 0.036	0.32 ^d^ ± 0.021	0.29 ^c^ ± 0.011	0.24 ^b^ ± 0.018	0.23 ^a^ ± 0.002	0.29 ^c^ ± 0.020
S + NPK + B	0.31 ^bc^ ± 0.007	0.30 ^b^ ± 0.019	0.27 ^a^ ± 0.040	0.32 ^c^ ± 0.013	0.27 ^b^ ± 0.012	0.27 ^b^ ± 0.016	0.23 ^a^ ± 0.016	0.32 ^c^ ± 0.010

S—soil without additives, S + F—soil with fungal strains, S + B—soil with bacterial strains, S + NPK—soil with NPK, S + NPK + F—soil with fungal strains and NPK, S + NPK + B—soil with bacterial strains and NPK.

**Table 4:** Annual fertilization plan for Abruptic Luvisol (AL) and Brunic Arenosol (BA) soils. The same scheme was used in the second year of the experiment. Doses of mineral fertilizers, and fungal and bacterial strains were calculated separately for each soil on the basis of soil properties and requirements for maize cultivation.

Variant	Description	Symbol	AL Soil (Doses per 1 ha)	BA Soil (Doses per 1 ha)
I	Soil without NPK fertilizers	S	No additives	No additives
II	Soil without NPK fertilizers, treated with fungal strains	S + F	30.5 kg (17 + 7.5 + 6) fungal strains	32 kg (17 + 8 + 7) fungal strains
III	Soil without NPK fertilizers, treated with bacterial strains	S + B	14 kg bacterial strains	14 kg bacterial strains
IV	Soil with NPK fertilizers	S + NPK	300 kg Polifoska 6 (18 kg N + 60 kg P_2_O_5_ + 90 kg K_2_O) 135 kg potassium salt (81 kg K_2_O) 442 kg (295 + 147) ammonium nitrate (100 + 50 kg N)	250 kg Polifoska 6, (15 kg N + 50 kg P_2_O_5_ + 75 kg K_2_O) 162 kg potassium salt (97 kg K_2_O), 295 + 147 kg ammonium nitrate (100 + 50 kg N)
V	Soil with NPK fertilizers and fungal strains	S + NPK + F	300 kg Polifoska 6 (18 kg N + 60 kg P_2_O_5 +_ 90 kg K_2_O) 135 kg potassium salt (81 kg K_2_O) 30.5 kg (17 + 7.5 + 6) fungal strains 442 kg (295 + 147) ammonium nitrate (100 + 50 kg N)	250 kg Polifoska 6 (15 kg N + 50 kg P_2_O_5_ + 75 kg K_2_O) 162 kg potassium salt (97 kg K_2_O) 32 kg (17 + 8 + 7) fungal strains 442 kg (295 + 147) ammonium nitrate (100 + 50 kg N)
VI	Soil with NPK fertilizers and bacterial strains	S + NPK + B	300 kg Polifoska 6 (18 kg N + 60 kg P_2_O_5 +_ 90 kg K_2_O) 135 kg potassium salt (81 kg K_2_O) 14 kg bacterial strains 442 kg (295 + 147) ammonium nitrate (100 + 50 kg N)	250 kg Polifoska 6 (15 kg N + 50 kg P_2_O_5_ + 75 kg K_2_O) 162 kg potassium salt (97 kg K_2_O) 14 kg bacterial strains 442 kg (295 + 147) ammonium nitrate (100 + 50 kg N)

## Data Availability

Not applicable.

## Sample Availability

Samples of the humic acids are available from the authors.
